# Assessing the Effects of Aging on Muscle Stiffness Using Shear Wave Elastography and Myotonometer

**DOI:** 10.3390/healthcare9121733

**Published:** 2021-12-14

**Authors:** Yerim Do, Prarthana Sanya Lall, Haneul Lee

**Affiliations:** Department of Physical Therapy, College of Health Science, Gachon University, 191 Hambangmoe-ro, Yeonsu-gu, Incheon 21936, Korea; doyr62@gachon.ac.kr (Y.D.); lallprarthana@gachon.ac.kr (P.S.L.)

**Keywords:** aging, muscle, myotonometer, shear wave elastography, stiffness

## Abstract

The current study investigated the differences in muscle stiffness between older and young adults at rest and during contraction. We also evaluated the differences in muscle stiffness assessments using a myotonometer (MyotonPRO) and shear wave elastography (SWE). Twenty-two older adults (mean age, 66.6 ± 1.6 years) and 23 young adults (mean age, 66.6 ± 1.6 years) participated in this study. Muscle stiffness of the tibialis anterior (TA) and medial gastrocnemius (MG) muscles at rest and during contraction were measured using SWE and the MyotonPRO. The stiffness increase rate (SIR) was also calculated to determine the absolute stiffness difference. The mean muscle stiffness of the TA and MG muscles was significantly lower in older adults than in young adults at rest and during contraction (*p* < 0.05). Similarly, the SIR values of the TA and MG were significantly lower in older adults than in young adults (*p* < 0.05). Our results indicate that both instruments could be used to quantify muscle stiffness changes and serve as a cornerstone for assessing aging-related losses in muscle function. Stiffness measures may help exercise professionals to develop an in-depth understanding of muscle impairment at the tissue level.

## 1. Introduction

The World Health Organization (WHO, Geneva, Switzerland) insists that as life expectancy has increased, a demographic change has occurred, i.e., the proportion of older adults in the world population has increased. However, an increased life expectancy does not mean that all older adults have a high quality of life [1]. Rather, most experience a decrease in muscle function with aging due to various changes in the musculoskeletal system that lead to a functional decrease in balance, gait speed, independence in daily living, and quality of life [1,2,3]. 

Muscle mass is reduced by approximately 10% in individuals in their 50s compared to those in their 30s or 40s due to the decreased number and size of muscle fibers, which causes muscle atrophy [4]. Both types of muscle fibers decrease with aging, but the ratio of type IIa muscle fibers shows a greater alteration, with more than a 50% loss between the ages of 20 and 75 years. Moreover, regeneration of the skeletal muscle can be limited due to the loss of satellite cells related to the reduction in type II fibers [3,4,5]. A decrease in muscle mass is associated with a loss of muscle strength. A decrease in muscle density with an increase in intermuscular fat, a decline in hormones such as testosterone, and low levels of physical activity with aging can also contribute to declining muscle strength [3,5]. Muscle strength peaks in an individual’s 20s or 30s, decreases 1.5% per year in one’s 50s to 60s, and decreases 3% per year thereafter [6,7]. A greater decline occurs in the lower extremities than in the upper extremities, creating a functional decrease in the ankle joint stabilizers such as the tibialis anterior (TA), gastrocnemius, and soleus [8,9,10,11]. The TA is an ankle stabilizer and support of the peroneus muscle that maintains balance and locomotion [12]. The medial gastrocnemius (MG) contributes to stabilizing the body position with the lateral gastrocnemius (LG) and soleus [13]. The gastrocnemius is more influential than the soleus in terms of the plantar flexion strength and balance ability reductions that occur with aging, contributing to an increased fall rate [14]. Stiffness is defined in physics as the applied force, equal to a constant, times the displacement in length, according to the Hooke’s Law [15], which insists that the amount of change in the length of an object is proportional to the force applied to it [16]. The elastic energy is stored when deformation occurs, and it is used when an object returns to its initial state. When it comes to the human body, stiffness can be explained in a wide range of contexts, from a single muscle fiber to the whole body. Muscle stiffness is the muscle’s ability to maintain its initial structure against external forces to prevent deformities [17]. Several studies have investigated the alterations of muscle stiffness due to aging; however, the results are conflicting. One study reported an increase in muscle stiffness due to an increase in collagen fibers, while other studies reported a decrease in muscle stiffness due to the impairment of the connective tissue support function and skeletal muscle fat infiltration known as myosteatosis [18,19]. Alfuraih et al. suggested that muscle stiffness in older adults is related with muscle mass, function, strength, and walking time [18].

Fall is a geriatric syndrome that can cause serious damage in older adults because it is related to fatal fractures [20]. It is known that 30–40% of people over the age of 65 years are at risk of falling [21]. As the rate of fall increases to 50% at 80 years of age, it is considered a serious social problem [22]. Age-related differences in strategies used to maintain posture control were reported in a previous study [12]. Young adults tend to increase their postural sway to gain more proprioceptive information from the leg muscles when they are exposed to postural instability. In contrast, older adults use a co-contraction strategy, involving the ankle-stabilizing muscles, to maintain their balance due to the fear of losing stability and reduced proprioceptive acuity [12,23,24]. Thus, the role of muscles in maintaining balance and locomotion is more important in older adults than in young adults, and the ankle-stabilizing muscles should be assessed to observe differences between ages to identify risk factors for falls [12]. 

Assessments of muscle stiffness have been used to estimate muscle force, the mechanical properties of tissue, pain, and fatigue, and stiffness has been measured using various instruments. 

Among them, the MyotonPRO and shear wave elastography (SWE) are commonly used to measure muscle stiffness [25,26]. The MyotonPRO is a portable hand-held device that provides a non-invasive assessment of the biomechanical properties of the skeletal muscles with a low clinical cost [26]. It requires less training for examiners but offers a limited measurable area compared to SWE [27,28]. SWE is non-invasive, requires a short examination time, and shows the degree of elasticity using colors [29]. It was recently used in the musculoskeletal system to provide information for the follow-up treatment of tendons, ligaments, muscles, and nerves [30,31,32,33]. Although assessing age-related differences in the gastrocnemius and Achilles tendon using SWE and MyotonPRO were previously reported, studies on TA stiffness are lacking [34]. Therefore, this study aimed to identify differences in muscle stiffness at rest and during contraction between older and young adults. We hypothesized that older adults have significantly lower muscle stiffness than young adults as measured using the MyotonPRO and SWE. 

## 2. Materials and Methods

### 2.1. Ethics Statement

The present study was conducted according to the Declaration of Helsinki and was approved by the Institutional Review Board of Gachon University (Seongnam, Korea) (1044396-201908-HR-139-01). The manuscript was prepared according to the Strengthening the Reporting of Observational Studies in Epidemiology guidelines [35]. All participants were asked to provide written informed consent before participating in the study. 

### 2.2. Participants

A total of 44 participants were included in the study, including 21 older adults and 23 young adults. The study’s purpose, procedure, and risks were explained to all subjects before the start of the study. Subjects were included if they had a body mass index of 18–25 kg/m^2^, were at least 60 years of age (older adults), and completed less than 150 min of physical activity of any intensity per week [27]. The subjects were excluded from participating if they had any musculoskeletal, orthopedic, neurological, cardiovascular disease, or current pain in the lower limbs. 

G·Power 3.1.9.7 software (Heinrich-Heine-University Dusseldorf, Düsseldorf, Germany) was used to determine the sample size. The probabilities of an alpha error and power were set at 0.05 and 0.95, respectively, with an effect size of d = 1.8 (*t*-test, difference between two independent means) based on a previous study [36]. Therefore, a sample size of 20 participants was required. With an estimated dropout rate of approximately 20%, 24 participants were needed to ensure statistical power.

### 2.3. Experimental Protocol

When the subjects arrived, they were asked to rest comfortably at room temperature for 10 min to stabilize their body conditions. Height, weight, and body mass index were measured before the start of the experiment. All subjects were asked to perform active voluntary contraction and were informed about how to contract each muscle correctly. The measurement area was exposed, and the exact location was checked and marked with a permanent marker. For cross-checking, the SWE transducer was placed transversely on the belly of both target muscles. The point of greatest muscle thickness was assessed in the image, and the area was marked on the skin for the measurements. For the SWE and MyotonPRO stiffness measurements, the subjects were asked to relax each target muscle for 30 s, during which the SWE and MyotonPRO measurements were taken, and then they contracted each target muscle as informed for 10 s for SWE and an additional 10 s for the MyotonPRO. A 30 s rest was provided between the two measurements. 

### 2.4. Measurement Position 

The largest cross-section of each muscle belly of the dominant leg was marked with a permanent marker for measuring stiffness from identical points for repeated measures of SWE and MyotonPRO. The assessment points and measurement positions used in our study were similar to those used in a previous study [27]. 

Tibialis anterior: To test the resting stiffness of the TA, the subjects were asked to lie supine. The stiffness of the contracted TA was assessed with 15° dorsiflexion of the foot.

Medial gastrocnemius: The subjects were positioned prone with their feet off the bed. The largest cross-sectional area of the muscle belly was identified as the measurement point. The subjects were asked to perform a 35° plantar flexion to measure the stiffness of the contracted MG.

### 2.5. Outcome Measurements

#### 2.5.1. Shear Wave Elastography

An ACUSON S3000 ultrasound device (Siemens Healthcare, Erlsangen, Germany) with a 9 MHz linear probe was used for the SWE measurements. SWE generates a color map overlay representing a qualitative elastogram and an objective measurement of stiffness. The elastographic images are produced with red and blue colors, with the red color indicating high tissue stiffness and the blue color indicating low stiffness using the SWE imaging technique [37]. The probe was placed parallel to the muscle fibers during the acquisition of data. It was positioned in a stationary manner with a coupling gel and very light pressure. Once the examiner found the thickest portion of belly muscle using B-mode imaging, SWE imaging was performed at that point. The stiffness values are represented in kilopascals (kPa). Previous studies demonstrated good intra-rater reliability for stiffness measurements of the lower limb muscles [27,38]. In addition, the stiffness increase rate (SIR) was calculated to determine the absolute stiffness difference as: (S_contracted_ − S_resting_)/S_resting_ [36]. 

#### 2.5.2. MyotonPRO

Mechanical measurements of muscle stiffness were measured using a MyotonPRO hand-held digital palpation device (Myoton AS, Tallin, Estonia). This device is highly reliable and valid, and it has also been used to measure superficial, soft biological tissue, and non-vital physiological parameters in research and clinical use [25,39,40,41]. The instrument’s probe was placed on the previously marked points of both target muscles. The impulse produced by the probe on the muscles lasts for 15 ms, with a light mechanical force of 0.6 N [42]. The force applied to the underlying tissue by the MyotonPRO creates dampened free oscillatory decay that is measured using the device. The device’s screen displays the calculated mechanical stiffness measurement in Newtons per meter. The MyotonPRO exhibited excellent to good reliability for the lower limb muscles [41,42]. 

### 2.6. Statistical Analysis

SPSS 26.0 software for Windows (IBM Corp., Armonk, NY, USA) was used to analyze the data. All data are represented as mean ± standard deviation (SD) and were tested for normal distribution using the Shapiro–Wilk test. An independent *t*-test was used to compare the muscle stiffnesses of young versus older adults. Then, effect size of Cohen’s d was used to identify how strong the mean difference between young and older adults. Pearson’s correlation was conducted to examine the association between muscle stiffness measured with the SWE and MyotonPRO in each muscle during the rest and contraction. The intra-rater reliability of the measurement was calculated using the intra-class correlation coefficient (ICC) with a two-way mixed effect model. The level of significance was set at α = 0.05.

## 3. Results

The mean age of the young adults was 22.5 ± 2.0 years, while that of the older adults was 66.6 ± 1.6 years. No significant differences were noted except for age. The participants’ general characteristics are listed in Table 1. 

### 3.1. Differences in Muscle Stiffness between Young and Older Adults

Table 2 describes the muscle stiffness and SIR in young and older adults. The mean muscle stiffness of the TA and MG was significantly lower at rest in older adults than in young adults in the SWE and the MyotonPRO (*p* < 0.05). In addition, a significant difference in muscle stiffness was found when the muscles were contracted, and the effect size was even greater than that observed at rest (*p* < 0.01).

There was a significant difference in SIR values of the TA and MG muscles measured using SWE between young and older adults (*p* < 0.05). However, a significant difference in the SIR value was not found in the TA (*p* > 0.05), but was found in the MG (*p* < 0.045) measured using MyotonPRO. 

### 3.2. SWE and MyotonPRO Muscle Stiffness Measurements of Young vs. Older Adults

For the overall population, strong correlations were found between the stiffness values of all muscles at rest using SWE and the MyotonPRO (MG: *r* = 0.471, *p* < 0.001; TA: *r* = 0.484, *p* < 0.001). Additionally, significant moderate correlations were observed when the muscles were contracted (MG: *r* = 0.605, *p* < 0.001; TA: *r* = 0.610, *p* < 0.05). 

In young adults, strong correlations were found between the stiffness values of all muscles at rest using SWE and the MyotonPRO (MG: *r* = 0.631, p < 0.001; TA: *r* = 0.561, *p* < 0.001). Additionally, significant moderate correlations were observed when the muscles were contracted (MG: *r* = 0.471, *p* < 0.05; TA: *r* = 0.636, *p* < 0.01). 

In the elderly population, there were significant correlations in stiffness during contraction in the MG but not the TA (MG: *r* = 0.466, *p* < 0.05; TA: *r* = 0.370, *p* > 0.05), while moderate to strong correlations were found in both muscles at rest (MG: *r* = 0.414, *p* = 0.050; TA: *r* = 0.482, *p* < 0.05).

### 3.3. Intra-Rater Reliabilities

#### 3.3.1. Shear Wave Elastography

For the overall population, the intra-rater reliabilities of the stiffness of TA and MG muscles measured with SWE were good to excellent. The ICCs were 0.906 (95% confidence interval (CI): 0.824–0.949) for TA and 0.981 (95% CI: 0.964–0.990) for MG at resting, and 0.851(95% CI: 0.723–0.920) for TA and 0.63 (95% CI: 0.746–0.927) for MG during contraction.

In young adults, the ICCs were 0.920 (95% CI: 0.811–0.966) for TA and 0.982 (95% CI: 0.957–0.992) for MG at resting, and 0.776 (95% CI: 0.471–0.905) for TA and 0.768 (95% CI: 0.399–0.911) during contraction.

In older adults, the ICCs were 0.897 (95% CI: 0.733–0.960) for TA and 0.966 (95% CI: 0.911–0.987) for MG at resting, and 0.840 (95% CI: 0.584–0.938) for TA and 0.819 (95% CI: 0.573–0.923) for MG during contraction.

#### 3.3.2. MyotonPRO

For the overall population, the intra-rater reliabilities of the stiffness of TA and MG muscles measured with MyotonPRO were good during rest. The ICCs were 0.945 (95% CI: 0.898–0.970) for TA and 0.923 (95% CI: 0.856–0.958) for MG at resting, and 0.668 (95% CI: 0.558–0.825) for TA and 0.595 (95% CI: 0.495–0.852) for MG during contraction.

In young adults, the ICCs were 0.933 (95% CI: 0.842–0.972) for TA and 0.893(95% CI: 0.747–0.955) for MG at resting, and 0.676 (95% CI: 0.404–0.915) for TA and 0.612 (95% CI: 0.352–0.841) during contraction.

In older adults, the ICCs were 0.934 (95% CI: 0.830–0.975) for TA and 0.924 (95% CI: 0.803–0.971) for MG at resting, and 0.635 (95% CI: 0.395–0.825) for TA and 0.545 (95% CI: 0.373–-0.883) for MG during contraction. 

## 4. Discussion

Decreased muscle mass and stiffness in older adults limit their balance function [12,18]. It is very important to assess muscle stiffness between young and older adults and identify their risk factors for falls. Therefore, the current study examined the stiffnesses of the ankle stabilizing muscles, including the TA and MG, using SWE and a myotonometer, and the main null hypothesis of our study was rejected as a result. Our study revealed that muscle stiffness in the elderly was lower than in young adults both at rest and during contraction. Our finding is consistent with the findings of previous studies, which showed that the muscle stiffness of older adults was relatively reduced [18,36,43]. However, unlike a previous study that reported no significant difference in the absolute stiffness difference in the medial head of the MG between older adults and younger adults [36], the SIR values of the TA and MG were significantly lower in elderly as compared to young adults in the current study. 

Aging is related to a continuous reduction in muscle mass, strength, and quality (sarcopenia). Muscle contraction occurs when the sarcomere is shortened by the cross-linking of myosin (thick filaments) and actin (thin filaments) in sarcomeres. When myosin and actin filaments are in a strong-binding structural state, force is generated; hence, age-related structural or chemical alterations in actin and myosin influence the weak-to-strong actomyosin transition. Specifically, there is an age-related decline in the contractility of single fibers. Previous researchers have stated that structural changes in myosin are linked with muscle fiber relaxation and contraction, which are in turn associated with an age-related decrease in force [44,45]. The total amount of force generated can be reduced because of structural changes in the skeletal muscles with aging [36]. According to a previous study, muscle contraction level and stiffness were positively correlated [46], which may indicate a relationship between stiffness and force generation. Our results also support that the force created by contraction is related to absolute stiffness.

Sendur et al. claimed that the reason that the SIR did not significantly differ was that structural changes related to skeletal muscle aging decreased the total amount of generated force, whereas the ability to generate force remains proportionally similar [36]. However, the force generated by muscle contraction was significantly lower in older adults than in younger adults. Our results showed that the difference in muscle stiffness between the age groups was greater during contraction than during rest. This indicates a smaller change in the absolute stiffness difference in the elderly adults because of the relatively greater decrease in force generation during contraction than at rest in older versus young adults. The difference in results from previous studies is probably due to the age range of the elderly population. In a previous study, the elderly group included participants over 50 years of age, which may not have sufficiently shown age-related changes [36]; however, in the current study, participants aged 60 years or older were included in the elderly population, and this age difference may have caused the different results. 

Moreover, objectifying one aspect of the muscle’s biomechanical state is a novel method for the quantitative measurement of muscle stiffness [26]. In the current study, we observed a good to excellent intra-rater reliabilities in muscle stiffness measured by the two instruments at rest, but the ICC was moderate to good during contraction in both young and older adults. 

In addition, we observed a moderate correlation in muscle stiffness measured by the two instruments at rest and during contraction, but this relationship was not strong in the TA muscle during contraction in older adults. Our results are comparable with those of several studies that reported moderate to strong associations between SWE and the MyotonPRO, but only in young adults. Feng et al. found a significant correlation between the resting stiffness of the gastrocnemius muscle and the Achilles tendon [25] while Kelly et al. assessed the correlation in the infraspinatus, erector spinae, and gastrocnemius muscles at various contraction intensities; the correlation was significant in all muscle regions but was strongest in the gastrocnemius muscle [26]. In addition, Lee et al. found a moderate to good correlation between these two instruments in the lower limb muscles at rest and during contraction [27]; however, to the best of our knowledge, no studies have been conducted in older populations. In the present study, the relationship was stronger in young adults than in older adults in the MG and TA at rest and under contraction, which is evident from the contractility of muscle fibers, the muscle mass, and the total amount that force generation decreases with aging due to structural changes in the skeletal muscles [44,46]. However, during contraction in older adults, the correlation between the two instruments was significant for the MG but not significant for the TA muscle. Muscle contraction level and stiffness have a positive relationship; therefore, the force generated by the TA was greater than that generated by the MG as the stiffness measures were higher for the TA. Our results show that the TA has more variation in stiffness measures between the rest and contraction states than the MG measured using the MyotonPRO and SWE, and this variation may have affected the overall correlation, especially in the elderly population. Furthermore, the MyotonPRO is susceptible to interference from the overlying skin and subcutaneous fat, whereas SWE is not [47]. However, correlation coefficient only measures the strength of linear correlation between the two sets of variables, but cannot measure the agreement of the two methods [48]. Since the measurement units of the two instruments are different and they are not convertible, a Bland–Altman plot, which measures the agreement of the two sets of data, was not used. The SWE measures the stiffness using the Young’s modulus and the compressive stiffness of a solid material, such as a soft tissue, when force is applied to the area and is represented as kilopascal (kPa), while the MyotonPRO measures the resistance to an external force that deforms the initial shape and is represented as Newton per meter (N/m). As the previous research mentioned, even though the term stiffness is commonly used to define the viscoelastic characteristics when assessing tissues, the two methods assess different types of stiffness [49].

In addition, it is important to indicate the metabolic alterations and their relation to changes in muscle with age as the metabolic characteristics are crucial predictors of muscle quality. Several inflammatory cytokines have been found to cause decreased muscle mass. The C-reactive protein, a marker of systemic inflammation, is directly related to age-related skeletal muscle deterioration. High levels of interleukin-6 are associated with an increased risk of muscle mass loss and decreased strength in older adults. On the other hand, increased levels of the tumor necrosis factor can stimulate catabolic pathways in skeletal muscles, eventually leading to sarcopenia [50]. Therefore, these factors might affect the results. Therefore, further studies should define whether these factors affect the stiffness of the ageing population.

The current study is the first to evaluate the correlation between measurements of the TA and MG muscles taken using SWE and the MyotonPRO at rest and during contraction in young versus older adults, but its limitations must be acknowledged. First, contraction intensity and position were not strictly controlled, and muscle force during contraction was not quantitatively measured. Differences in voluntary activation levels during contraction may have influenced the stiffness measurements. Second, the older adults who participated in this study were over 60 years of age. According to WHO standards, older adults are aged 65 years or older [1]. Although the mean age of the older adults in this study was 66.6 years, they were still not old enough, so further studies should include an older population (≥70 years) to more accurately compare the characteristics of older adults. In addition to the stiffness measurement, assessing functional outcomes for predicting the risk of falls, such as static and dynamic balance ability, postural sway, or gait analysis, are necessary to validate the results of the current study. If lower muscle stiffness in the elderly population is related to these characteristics, then, as previously reported [18], a lower muscle stiffness can be considered a risk factor for falls.

## 5. Conclusions

Our results indicate that muscle stiffness measured using both instruments was significantly lower in older adults than in young adults both at rest and during contraction, while the SIR was significantly lower in the elderly adults. These findings suggest that both instruments could be used to quantify muscle stiffness changes and serve as a cornerstone for assessing aging-related losses in muscle function. Moreover, stiffness measures may help exercise professionals to develop an in-depth understanding of muscle impairment at the tissue level, and eventually they may be used to design effective exercise regimes that focus on preventing muscle loss and other metabolic muscular alterations that occur with age.

## Figures and Tables

**Table 1:** General participant characteristics (n = 44).

	Young Adults (n = 23)	Older Adults (n = 21)
Age (years)	22.5 ± 2.0	66.6 ± 1.6
Female sex, n (%)	11 (47.8)	9 (42.9)
Height (cm)	165.2 ± 7.6	163.1 ± 9.4
Weight (kg)	60.1 ± 10.4	62.9 ± 10.7
Body mass index (kg/m^2^)	21.8 ± 2.4	23.6 ± 2.8

**Table 2:** Difference in muscle stiffness between young and older adults (N = 44).

Muscle		SWE (kPa) (Mean ± SD)			MyotonPRO (N/m) (Mean ± SD)		
		Young (n = 23)	Older (n = 21)	*d ^†^*	Young (n = 23)	Older (n = 21)	*d ^†^*
Tibialis Anterior	R	20.2 ± 1.6	18.2 ± 1.4 **	1.06	349.6 ± 27.5	325.5 ± 25.7 **	0.90
	C	140.9 ± 24.2	115.7 ± 26.5 **	1.13	797.0 ± 80.1	705.2 ± 48.1 **	1.35
	SIR	6.04 ± 1.34	5.16 ± 1.20 *	0.68	1.29 ± 0.29	1.18 ± 0.21	0.44
Medial Gastrocnemius	R	12.3 ± 1.8	10.8 ± 1.3 **	0.93	258.6 ± 14.6	245.8 ± 18.1 *	0.78
	C	90.2 ± 12.2	74.0 ± 11.5 **	1.36	384.5 ± 44.2	338.2 ± 35.8 **	1.14
	SIR	6.45 ± 1.25	5.93 ± 1.29 *	0.41	0.49 ± 0.17	0.38 ± 0.16 *	0.65

Abbreviation: R—resting; C—contraction; SIR—stiffness increase rate, ***^†^*** cohen’s d; effect size for the comparison between two means, ** significant difference between young and old adults at the 0.01 level, * significant difference between young and old adults at the 0.05 level.

## Data Availability

The datasets generated during this study are available from the corresponding author upon reasonable request.
